# Effect of anaesthetic technique on neonatal morbidity in emergency caesarean section for foetal distress

**DOI:** 10.1371/journal.pone.0207388

**Published:** 2018-11-16

**Authors:** Ipek Saadet Edipoglu, Fatma Celik, Elif Cirakoglu Marangoz, Gulin Haroglu Orcan

**Affiliations:** 1 Department of Anesthesiology, Istanbul Training and Research Hospital, Istanbul, Turkey; 2 Department of Anesthesiology, Ergani Government Hospital, Diyarbakır, Turkey; 3 Department of Anesthesiology, Mardin Birth and Women's Health Education and Research Hospital, Mardin, Turkey; University of Bari, ITALY

## Abstract

**Background:**

While foetal distress is typically associated with ischaemic injury, few studies have assessed neonatal morbidity for emergency caesarean section. Moreover, the decision of the anaesthetic technique may be of paramount importance in emergency caesareans, because of the limited time and increased risk. We aimed to evaluate the effect of the anaesthetic technique on neonatal morbidity in emergency caesarean indicated for foetal distress.

**Methods:**

This was a single-centre, prospective observational study, conducted between July-2015 and December-2015. The study enrolled parturient with indication for emergency caesarean section after diagnosis of foetal distress, who received either regional or general anaesthesia. The outcome measures were: 1, 5-minute Apgar scores; umbilical blood pH; length of hospitalization; and morbidity, defined as a 5-minute Apgar score <7, need for mechanical ventilation, admittance to a neonatal intensive care unit, or respiratory insufficiency symptoms.

**Results:**

61 patients were included in the study, of whom 31 received regional anaesthesia. Neonatal morbidity was noted in 5 and 9 cases with regional and general anaesthesia, respectively. The 1-minute Apgar score was significantly lower(*p* = 0,045) for cases with general anaesthesia, which was not true for the 5-minute Apgar score. Regional anaesthesia was non-significantly associated with shorter length of hospitalization, lower incidence of morbidity, and higher umbilical blood pH. When we take regional anaesthesia cases as a reference point, we detected that general anaesthesia cases are showing 2,2 times more morbidity risk. But these results did not reach any statistically significant levels.

**Conclusions:**

While we did find some improved results for regional anaesthesia group, we found no statistical evidence that neither anaesthesia technique is superior regarding neonatal morbidity. We think that regional anaesthesia should be preferred whenever possible because of our improved results of length of hospital stay, APGAR and morbidity and we think that general anaesthesia is indicated for very urgent cases or regional anaesthesia contraindicated patients.

**Trial registration:**

http://www.isrctn.com/ISRCTN15181117

## Introduction

Cases with emergency caesarean section are challenging for the obstetric anaesthetist, because such cases are typically associated with life-threatening conditions for the foetus or the mother. For women requiring emergency caesarean section, a 30-minute decision-to-delivery interval (DDI) for caesarean section has become the standard target in clinical practice, although DDIs of 30–75 minutes have been proposed [1]. The guidelines put forward by the Royal College of Obstetricians and Gynaecologists (RCOG) suggest that caesarean section should be carried out “with an urgency appropriate to the risk to the baby and the safety of the mother”, as soon as the target DDI is established [1]. Given the limited time and increased risk, both maternal and foetal outcome depend on the coordination and vigilance of the anaesthetist, and the choice of anaesthetic technique is of paramount importance. For elective caesarean section, regional anaesthesia is recommended over general anaesthesia, as there is less aspiration risk with regional anaesthesia, and no risk for intubation-related complications [2]. Moreover, general anaesthesia may depress the Apgar 1-minute score of the new-born. [3–5] For emergency caesarean section, regional anaesthesia can be applied only when there is sufficient time [6,7]. Regional anaesthesia is the preferred anaesthetic technique for the majority of caesarean sections performed in many hospitals. While many emergency caesarean sections are performed each year, limited data are available regarding the best anaesthetic practise for cases with foetal distress. Foetal asphyxia, typically referred to as foetal distress, can lead to ischaemic injury. Compensatory mechanisms involving the redistribution of blood to vital organs enable the foetus to survive asphyxia, provided that the duration of asphyxia is limited [6,7]. Few studies have investigated neonatal morbidity related to foetal distress diagnosed in cases with emergency caesarean section.

We conducted the present study to identify the most suitable anaesthetic technique for cases with foetal distress, and thus provide optimum care to the neonates born in our hospital. The primary aim of our study was to investigate the effect of the anaesthetic technique (i.e., regional or general anaesthesia) on neonatal morbidity in emergency caesareans. Length of hospitalization, and umbilical cord pH values were secondary outcomes.

## Materials and methods

Our single centre, prospective observational study was approved by the Ethical Committee for Clinical Studies of Bakirkoy Dr. Sadi Konuk Education and Research Hospital (study protocol code: 2015/127). The study was registered with the ISRCTN registry (ISRCTN) under the study ID ISRCTN15181117 (“The effect of anaesthetic technique on neonatal morbidity in emergent caesarean section for foetal distress”). The trial was hosted at tertiary education and research hospital. The trial had a duration, from 01/07/2015 to 06/12/2015. All study subjects provided written informed consent for participating in the trial.

The study enrolled pregnant women based on the following inclusion criteria: diagnosis of foetal distress; and age, 18–45 years. The following exclusion criteria were applied: neurological disease; body mass index ≥ 40 kg/m^2^; and sensitivity to any of the drugs used during the emergency caesarean section procedure. Each woman received either regional or general anaesthesia, and was thus included in group R or group G, respectively. All subjects underwent preoperative evaluation, physical examination, laboratory investigations (haemoglobin levels, platelet count, prothrombin time, blood glucose levels, and serum creatinine levels), and assessment of physical status class according to the American Society of Anaesthesiologists classification. For the decision of the anaesthetic approach, we used ‘patient preference’. We graded the urgency of cases according to RCOG guidelines classification [1]. Category 1: Maternal or foetal compromise and immediate threat to life of woman or foetus; Category 2: Maternal or foetal compromise and no immediate threat to life of woman or foetus; Category 3: No maternal or foetal compromise and requires early delivery; Category 4: No maternal or foetal compromise and can be done at a time to suit the woman and maternity services [1]. Patients had the anaesthetic approach they preferred, for all patients with sufficient time for the decision of the technique (risk classification category 2,3,4). For patients with insufficient time (category 1) to use a neuraxial anaesthetic or have contraindications for regional or general anaesthesia, we informed the patient and we inclined the patient for the indicated approach. We defined, unfasted patients and known prior difficult intubation anamnesis for contradiction for general anaesthesia. We defined contradiction for regional anaesthesia as bleeding disorder, infection in the intervention site and intracranial pathologies.

Foetal distress diagnosis and indication for emergency caesarean section were established by the same senior obstetrician for this study. And all the postoperative interventions were followed by the same obstetrician. Informed consent was taken by a resident as soon as the senior obstetrician decided to manage the caesarean section. During the caesarean section procedure, all patients were monitored via electrocardiographic measurements, as well as via non-invasive measurements of arterial blood pressure and oxygen saturation. As this was an observational study, we did not make any changes to the anaesthetic technique applied in our institution. Routine general anaesthesia induction for emergency caesarean involved pre-oxygenation using 100% oxygen, followed by a rapid sequence induction with propofol (2 mg kg^-1^) and rocuronium (0.9 mg kg^-1^). Anaesthesia was maintained with sevoflurane (2%) and a mix of 50% O_2_ and 50% nitrous oxide. Routine regional anaesthesia administration involved a rapid, single shot of 2.2 ml hyperbaric bupivacaine (0.5%) into the subarachnoid space of the L3-L4 intervertebral space. If hypotension was detected (mean arterial pressure, <60 mm Hg), 500 ml of colloidal solution were administered, along with ephedrine (5 mg, single dose) if hypotension persisted.

The following data were recorded: demographic data; decision-to-delivery interval; time of anaesthetic induction; location of incision to skin; delivery time (from skin incision to delivery of the baby); duration of operation (from skin incision to skin closure); intra-operative heart rate; intra-operative mean arterial pressure; 1, 5-minute Apgar scores, recorded by a neonatologist; complications noted immediately after birth, such as umbilical blood gas; follow-up until discharge; and morbid conditions. We defined neonatal morbidity as new-borns who had one or more of the following outcomes a 5-minute Apgar score of <7, the need for mechanical ventilation, admittance to a neonatal intensive care unit (NICU), or symptoms of respiratory insufficiency. The following measurements were considered as secondary outcomes in the present study: 1-and 5-minute Apgar scores; umbilical blood pH; length of hospital stay (LOS); and morbidity.

Patient sample analysis was made by Power and Sample Size Program (P. S version 3.1.2). The sample size was calculated on the root of our preliminary experimental results. We assumed that 58 patients were necessary when considering a %20 difference with α = 0.05 and power = 0.80 to be clinically significant based on our an umbilical blood pH results. We added %10 dropouts for each group and enrolled 70 patients for the analysis.

### Statistical analyses

All analyses were performed using NCSS (Number Cruncher Statistical System) 2007 (Kaysville, Utah, USA) program. Data were evaluated in terms of descriptive statistics including average, standard deviation, median, interquartile range (IQR) and frequency. Quantitative comparisons between groups were performed using the Mann Whitney U-test for non-normal distributions. To evaluate the comparison between anesthesia types and neonatal morbidity; the Yates Continuity correction test, which is a correction of Pearson Chi square test, was used. We tested assumptions of logistic regression and reported odds ratio (OR) with corresponding 95% confidence intervals (CI). Statistical significance was defined as *p* < 0.05.

## Results

A total of 70 women were evaluated, of whom 9 were not included in the study because they refused to participate. A total of 61 patients were included in the study (S1 Fig- flow diagram), of whom 31 received regional anaesthesia (group R) and 30 received general anaesthesia (group G). We did not note any differences between the groups in terms of demographic data (Table 1). (p>0,05)

**Table 1:** Demographic data of parturient.

	Regional anaesthesia (n = 31)	General anaesthesia (n = 30)	*p*-value
**Age (years)**	26.26 ± 5.36	26.90 ± 5.39	0.647
**BMI (kg/m**^**2**^**)**	28.48 ±2.45	28.69 ± 3.39	0.788
**Gestation (weeks)**	38.20 ± 2.97	37.89 ± 2.20	0.658
**Operation time (min)**	28.94 ± 8.61	30.67 ± 16.75	0.612
**Decision to delivery interval (min)**	21.50 ± 4.82	20.48 ± 3.79	0.363

*Mann Whitney U-test*. *Data given as mean ± standard deviation*. *p-values of <0*,*05 were considered significant*. *BMI*: *body mass index min*:*minutes*

In terms of operative indication as expected, non-reassuring foetal heart rate was the leading cause of indication for emergency caesarean section (n = 38; 62.2%). Late deceleration and foetal bradycardia were the two main causes with 17 patients for each. (Table 2)

**Table 2:** Operative indication for emergency caesarean section.

	Regional anaesthesia n = 31(%)	General anaesthesia n = 30(%)
**Non-reassuring foetal heart rate**		
*Late deceleration*	9 (29.0)	8 (26.6)
*Foetal bradycardia*	8 (25.8)	9 (30.0)
*Foetal tachycardia*	1 (3.2)	2 (6.6)
*Variable deceleration*	1 (3.2)	NA
**Meconium**	3 (9.6)	2 (6.6)
**Uteroplacental insufficiency**	3 (9.6)	2 (6.6)
**Placenta previa**	1 (3.2)	NA
**Abruptio placenta**	1 (3.2)	NA
**Oligohydramnios**	3 (9.6)	1 (3.3)
**Malpresentation**	NA	2 (6.6)
**Umbilical cord prolapses**	1 (3.2)	1 (3.3)
**Premature membrane rupture**	NA	1 (3.3)
**HELLP syndrome**	NA	1 (3.3)
**Foetal anomaly**	NA	1 (3.3)

*Data given as total number (%percentage)*.

When we evaluate the neonatal morbidity, we have seen 14 morbid cases. In group R, morbidity was noted in 5 (16.1%) neonates, whereas in group G (n = 30) neonate morbidity was detected for 9 (30%) cases; the difference was not statistically significant (Table 3). (p>0,05). When we take regional anaesthesia cases as a reference point, we detected that general anaesthesia cases are showing 2,2 times more morbidity risk. (OR = 2,229 (%95 CI: 0,648–7,664). (Table 3) But these results did not each any statistically significant levels. (p>0,05) 11 (%78.6) patients transferred to NICU and 4 patients (%28.5) had respiratory distress. 9 (%64.2) had 7<APGAR. All patients transferred to NICU necessitate mechanical ventilation

**Table 3:** Association of neonatal morbidity and anesthesia type.

		Neonatal Morbidity (-) (n = 47)	Neonatal Morbidity (+) (n = 14)	*p*
**Anesthesia type** *n(%)*	*Regional anaesthesia*	26 (83,9%)	5 (16,1%)	^***b***^***0*,*325***
	*General anaesthesia*	21 (70,0%)	9 (30,0%)	

*bYates Continuity Correction*. *p-values of <0*,*05 were considered significant*

Furthermore, group R exhibited a significant reduced heart rate for the mother compared to group G, measured after 10, 20, and 30 minutes from the start of the procedure (*p* = 0.032, *p* = 0,001, and *p* = 0.034 respectively; Table 4).

**Table 4:** Haemodynamic data of women indicated for emergency caesarean section.

	Regional anaesthesia (n = 31)	General anaesthesia (n = 30)	*p*-value
**MAP initial- 0 time (mmHg)**	98.58 ± 11.76	105.46 ± 17.03	0.073
**MAP at 10 minutes (mmHg)**	82.67 ± 12.97	91.03 ± 18.74	0.470
**MAP at 20 minutes (mmHg)**	83.09 ± 12.50	86.10 ± 18.10	0.453
**MAP at 30 minutes (mmHg)**	84.00 ± 8.47	89.68 ± 10.39	0.124
**HR initial– 0 time (beats/min)**	102.97 ± 19.44	105.90 ± 14.87	0.517
**HR at 10 minutes (beats/min)**	94.65 ± 16.96	103.21 ± 12.67	0.032*
**HR at 20 minutes (beats/min)**	88.50 ± 11.50	99.76 ± 13.22	0.001*
**HR at 30 minutes (beats/min)**	84.54 ± 9.43	92.13 ± 8.50	0.034*

*Mann Whitney U-test*. *Data given as mean ± standard deviation*.

*p-values of <0,*05 were considered significant*. *MAP*: *mean arterial pressure; HR*: *heart rate*

We also did not detect any significant difference in terms of LOS, although shorter LOS was found for group R. Similarly, the higher pH levels noted for group R did not amount to a statistically significant difference. On the other hand, we found significantly lower 1-minute Apgar scores (*p* = 0.045) in group G, though the differences between groups did not reach statistical significance for the 5-minute Apgar scores (*p* > 0.05; Table 5). The mortality rates were 1/31 and 2/30 for groups R an G respectively (p>0.05). One neonate from group R was lost to follow-up because the patient was transferred to another NICU.

**Table 5:** Neonatal information.

	Regional anaesthesia (n = 31)	General anaesthesia (n = 30)	*p*-value
**LOS (days)**	4.74 ± 1.61	6.16 ± 4.11	0.078
**1-minute Apgar (mean±SD)**	6.84 ± 1.65	6.07 ± 1.61	0.045*
**median (IQR)**	7 (6,8)	6 (3,6)	
**5**-**minute Apgar (mean±SD)**	8.55 ± 1.92	8.45 ± 1.52	0.458
**median (IQR)**	9 (7,9)	9 (6,7)	
**Umbilical Blood pH**	7.29 ± 0.15	7.20 ± 0.21	0.245
**Base Excess**	-5.33 ± 3.87	-6.19 ± 6.81	0.441

*Mann Whitney U-test*. *Data given as mean ± standard deviation*. *Length of hospitalization*: *LOS*

*p-values of <0,*05 were considered significant*. *interquartile range*: *IQR*

## Discussion

We believe that regional anaesthesia is preferred in elective caesarean sections because of the lower risk of complications, as reported in the literature [2,8,9]. Nevertheless, our results suggested that attending anaesthesiologists can use either techniques when managing foetal distress via emergency caesarean sections; specifically, although general anaesthesia was associated with lower 1-minute Apgar score and lower pH, the morbidity was similar to that found for regional anaesthesia. Thus, general anaesthesia is a more suitable option if there is very limited time for administering anaesthesia, or the mother presents with coagulopathy.

General anaesthesia can depress the Apgar score, although this is reversible and often not significant for the 5-minute scores [3–5]. Our findings are concurrent with the literature, as we detected significantly lower 1-minute Apgar scores for general anaesthesia, but no such difference was reflected in the 5-minute Apgar scores or morbidity. This behaviour is likely related to the fact that the duration of the exposure of the neonate to anaesthetic drugs is very brief, and thus the effect on the neonate remains visible only in the very first minute after birth [3]. In this context, regional anaesthesia can be viewed as having less effect on the neonate, though still resulting from physiological or biochemical changes in the mother [3].

In their database review, Algert and co-workers reported that, among the infants who did require intubation, a 5-minute Apgar score of <7 was more common in those that had been delivered with general anaesthesia than in those delivered with regional anaesthesia [2]. In our analysis, we did not find any differences between the groups in terms of intubation rates and 5-minute Apgar scores. The discrepancies most probably originate from patient selection strategies, as Algert and co-workers excluded pregnancies with reported hypertension, oligohydramnios, polyhydramnios, antepartum haemorrhage, and suspected foetal abnormality, reducing the risk for adverse events.

On the other hand, Saygı and co-workers evaluated the outcome of elective caesarean sections, and also found reduced 1-minute Apgar scores that did not reflect in the 5-minute Apgar scores or morbidity for regional anaesthesia group [10]. Jenniskens and Janssen reported that a 1-minute Apgar score of <7 after caesarean section is a good predictor of neonatal morbidity in infants with non-reassuring foetal status [11]. However, our results differ in terms of Apgar scores. There are further studies that reported no differences in the 1- and 5-minute Apgar scores between cases with general and those with regional anaesthesia [12].

When we evaluated regional anaesthesia cases as a reference point, we detected that general anaesthesia cases are showing 2,2 times higher morbidity risk. While we did note shorter LOS and lower morbidity in group R than in group G, the differences did not reach statistical significance. (*p* > 0.05). We detected that neither anaesthetic technique is statistically superior in terms of neonatal morbidity in emergency caesareans indicated for foetal distress. But we think that regional anaesthesia should be preferred whenever possible because of our improved results of LOS, APGAR and morbidity. As, there is substantial evidence in the literature for the higher risk of complications associated with general anaesthesia in caesarean section, thereby suggesting that administering regional anaesthesia is preferable, as there is no risk for difficult intubation and aspiration of gastric content [2,8,9,11,12]. Regional anaesthesia is unlikely to affect the neonate systemically because the doses of drugs being used for spinal anaesthesia are very low, and the time until delivery is typically short. However, in their review of 29 studies (1793 cases) registered in the Cochrane Database, Afolabi and co-workers also reported that there is no evidence that regional or general anaesthesia is superior in terms of outcomes of the caesarean section [13]. Another retrospective analysis of maternal and neonatal outcome after caesarean section did not demonstrate any significant difference between general and regional anaesthesia in terms of neonatal outcome [14].

Hillemanns and co-workers performed a retrospective analysis of 109 crash emergency investigations and reported that short DDIs (20–30 minutes) are associated with improved outcome, higher umbilical cord blood pH, and higher Apgar scores [15]. Our DDIs were also between 20–30 minutes for both groups. Similar to our findings, a recent study comparing foetal and maternal outcome in preeclampsia cases undergoing emergency caesarean section did not find a significant reduction in LOS associated with the anaesthetic technique [16]. While we did find shorter LOS in group R, the difference was not statistically significant.

Numerous studies have evaluated neonatal acid-base status and its association with the anaesthesia technique applied [17]. In their meta-analysis, Malin, Morris, and Khan reported that the low arterial pH was consistently associated with neonatal outcomes [18]. Strouch and co-workers evaluated 647 cases of emergency caesarean section and found significantly lower umbilical blood pH associated with the use of general anaesthesia (pH = 7.16) compared with those found for spinal anaesthesia (pH = 7.24) [17]. We found a similar trend regarding umbilical blood pH, with general anaesthesia being associated with lower pH values (7.20 versus 7.29 for regional anaesthesia), although this difference was not statistically significant (*p* > 0.05). On the other hand, Reynolds and Seed reported a lower umbilical pH for preterm infants in cases with spinal anaesthesia and attributed it to the usage of larger doses of ephedrine [3]. Strouch and co-workers suggested that the usage of phenylephrine instead of ephedrine may be responsible for the higher pH values noted in their study for spinal anaesthesia [17]. As we also used the minimal dose of ephedrine, we believe that this may explain the slightly higher pH values we observed for spinal anaesthesia.

A noteworthy study comparing the outcomes of spinal and general anaesthesia for caesarean section in preeclampsia patients reported a higher mean umbilical blood pH, which was attributed to hypocapnia induced by maternal hyperventilation and modest hypotension that may occur in mothers who receive spinal anaesthesia [19]. Although we did note a minor reduction in arterial pressure in group R, our patients received colloidal infusion and low-dose ephedrine when necessary, which helped avoid adverse events of hypotension. There is solid evidence that the duration and severity of maternal hypotension in caesarean section affects neonatal mortality and morbidity [10,20,21]. However, despite the fact that hypotension occurs frequently during caesarean sections, infants seem to tolerate this alteration in placental blood perfusion without any morbidity provided that fluid and inotropes are administered as appropriate [10,20].

Bradycardia of various degrees can be seen during caesarean sections when neuraxial anaesthesia is administered, and commonly results in a decrease in blood pressure [22,23,24]. The typical frequency of bradycardia was reported at 8%-15%, although Chamchad and co-workers detected bradycardia in 17% of the patients in their placebo group [22,23,24]. We also noted bradycardia in our neonates. The mechanisms responsible for bradycardia are diminished venous return and inhibition of sympathetic nerves [25].

The main limitation of our study is its prospective observational design. Specifically, we could not randomize the patients because we followed the appropriate medical indication for emergency caesareans. Furthermore, our results come from single-centre observations that may not be generalizable to all hospitals. In the future, a multicentre study with a larger study population is warranted to confirm our findings.

While we did find better results in terms of length of hospitalization and pH levels for women who received regional anaesthesia than for those who received general anaesthesia, these differences did not reach statistical significance. The 1-minute Apgar score was significantly lower when general anaesthesia was applied, which is concurrent with the literature. Overall, we found no statistical evidence that neither anaesthesia technique is superior regarding neonatal morbidity.

We think that regional anaesthesia should be preferred whenever possible because of our improved results of LOS, APGAR and morbidity and we think that general anaesthesia is indicated for very urgent cases or regional anaesthesia contraindicated patients.

## Supporting information

S1 Fig Flow chart of patient inclusion. (TIF)

S1 File CONSORT 2010 checklist. (DOC)

S2 File Duzenli etik kurul formlar fetal distres. (DOC)

S3 File Trial protocol in english. (PDF)
