# Supplementary figures and images for: Effect of anaesthetic technique on neonatal morbidity in emergency caesarean section for foetal distress
Source: PLoS One. 2018 Nov 16;13(11):e0207388. doi: 10.1371/journal.pone.0207388 (PMC6239306; doi:10.1371/journal.pone.0207388)

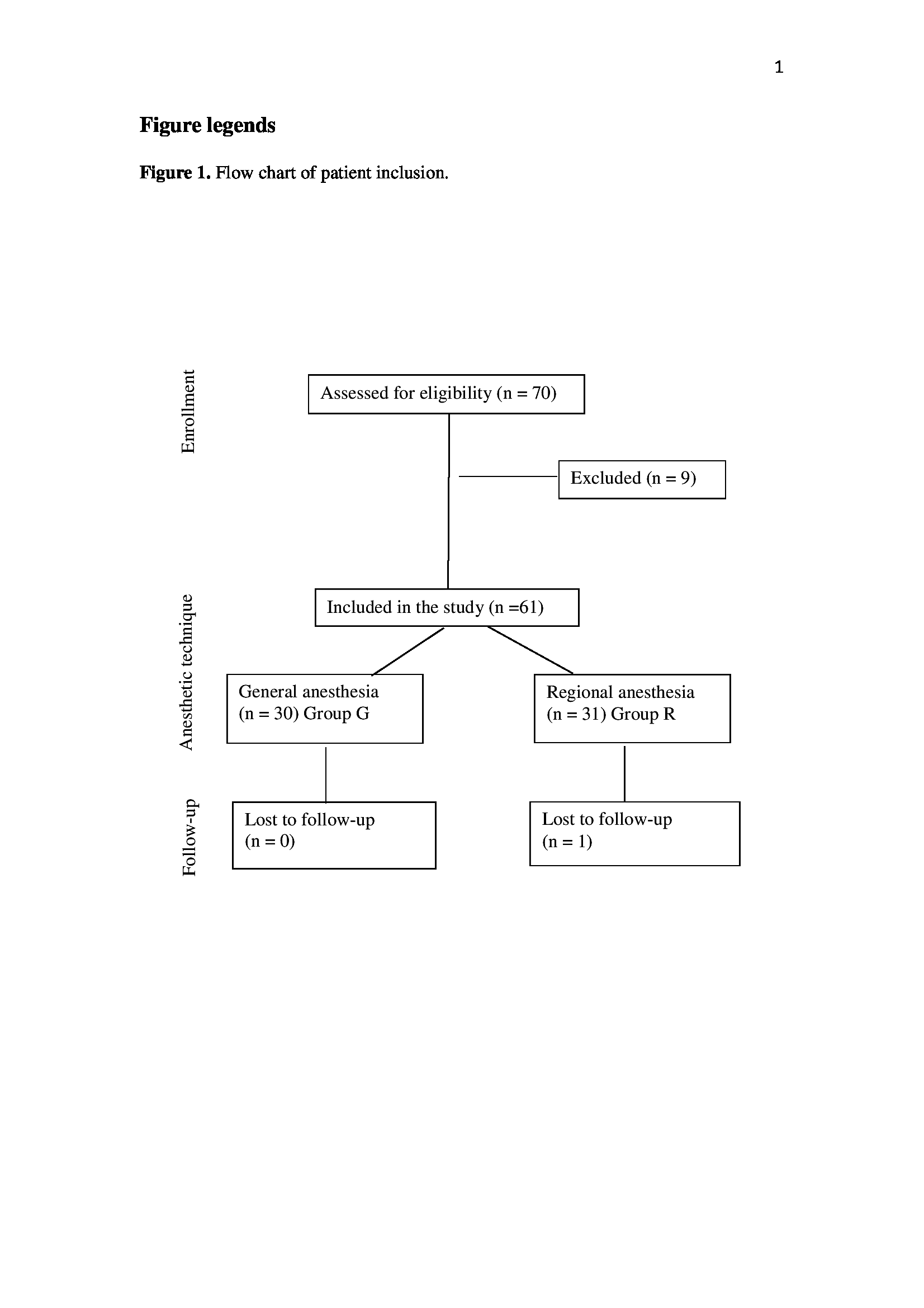

Supplement: S1 Fig — (TIF) [file pone.0207388.s001.tif]
