# Supplementary material for: Effect of anaesthetic technique on neonatal morbidity in emergency caesarean section for foetal distress
Source: PLoS One. 2018 Nov 16;13(11):e0207388. doi: 10.1371/journal.pone.0207388 (PMC6239306; doi:10.1371/journal.pone.0207388)
Supplement: S2 File — (DOC) [file pone.0207388.s003.doc]

**BAKIRKÖY DR. SADİ KONUK EĞİTİM ARAŞTIRMA HASTANESİ**

**KLİNİK ARAŞTIRMALAR ETİK KURULU BAŞVURUSU İSTENİLEN BELGELER** (BAKANLIK İZNİ KAPSAMI DIŞINDA KALAN BAŞVURULAR İÇİNDİR)

| ***BELGELER*** |
| --- |
| **Ön Bilgi Formu:** Araştırmacının tanımlayıcı bilgilerinin yer aldığı formdur. Etik Kurul sayfası Duyurular bölümünde ön başvuru formunu bulabilirsiniz. |
| **Başvuru Formu:** İEGM sitesi [www.iegm.gov.tr](http://www.iegm.gov.tr/) den başvuru formuna ulaşabilirsiniz. |
| **Araştırma Protokolü:** Klinik araştırmanın amacını, tasarımını, uygulanacak istatistiksel yöntemleri ve araştırmaya ait düzenlemeleri detaylı olarak tanımlayan belgedir. Dosyanızda bulunması gerkmektedir. |
| **Bilgilendirilmiş Gönüllü Olur Formu Örneği:** Gönüllünün belirli bir araştırmaya katılma kararıyla ilişkili olan araştırma hakkındaki bütün unsurlar konusunda bilgilendirildikten sonra, gönüllünün araştırmaya katılma konusunda istekliliğini kendi rızasıyla onayladığı süreç. Bilgilendirilmiş olur, yazılı, imzalı ve tarihli bilgilendirilmiş olur formu yoluyla belgelendirilmektedir. İmzasız taslağının dosyanınızda bulunması gerekmektedir. İEGM sitesi [www.iegm.gov.tr](http://www.iegm.gov.tr/) den BGOFformuna ulaşabilirsiniz. |
| **Olgu Rapor Formu Örneği:** Araştırmadaki her bir gönüllüye ait verilerin ve diğer bilgilerin protokolde tanımlandığı şekilde kaydının yapılması için hazırlanan basılı, optik veya elektronik belgedir. Dosyanızda bulunması gerekmektedir. |
| **Varsa, Araştırma Broşürü:** Araştırma ürünü veya ürünlerine ait klinik ve klinik olmayan verilere ait belgelerdir Araştırılan ürün veya ürünlere ait klinik ve klinik olmayan verilere ait belgelerdir. Varsa dosyanıza eklenmesi gerekmektedir. |
| **Araştırma Bütçe Formu:** İEGM sitesi [www.iegm.gov.tr](http://www.iegm.gov.tr/) den bütçe formuna ulaşabilirsiniz. |
| **Varsa, Sigorta** |
| **Varsa, Sözleşmeler** |
| **Literatür:** |
| **Başvuru Dosyasının CD Kaydı** |
| - Etik Kurula Başvuru dilekçesi |
| - İndeks → Dosya İçerik bilgisi (sayfalar numaralandırılmalı ya da ek olarak belirtilmelidir.) |
| - Başvuruya getirilen belgelerin sorumlu araştırmacı tarafından her sayfası paraf edilmelidir. |
| - Araştırmaya katılan Tüm Katılımcıların Öz geçmiş formlarının olması gerekmektedir.   Öz geçmiş formuna İEGM sitesi [www.iegm.gov.tr](http://www.iegm.gov.tr/) den ulaşabilirsiniz. |
| - Araştırma Dosyası Bilgisayar çıktılı olmalıdır. |
| - Dosyanızı Etik Kurul Toplantı gününden 5 önce etik kurul Sekreterliğine teslim etmeniz gerekmektedir. |

| 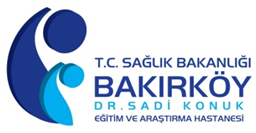   |  | | --- | |  |  |  |  |  |  |  |  |  |
| --- | --- | --- | --- | --- | --- | --- | --- | --- | --- | --- |
|  |  |  |  | Sorumlu Araştırmacının Adı: Saadet İpek Edipoğlu | | |  |  |  |
|  |  |  |  | Protokol No: | | |  |  |  |
|  |  |  |  | 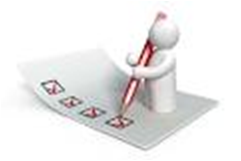 | | | |  |  |
|  |  |  |  |  |  |
|  |  |  |  |  |  |
|  |  |  |  |  |  |
|  |  |  |  |  |  |
|  |  |  |  |  |  |
|  |  |  |  |  |  |  |  |  |  |
| Klinik Araştırmalar Etik Kurulu İstenen Belgeler CheckListi | | | | | | | | | |
|  |  |  |  |  |  |  |  |  |  |
| 1) | Ön Bilgi Formu | | |  |  |  |  |  |  |
| 2) | Başvuru Formu | | |  |  |  |  |  |  |
| 3) | Her Sayfada İmza | | |  |  |  |  |  |  |
| 4) | Araştırma Protokolü | | |  |  |  |  |  |  |
|  | a) | Araştırmanın Yeri | | |  |  |  |  |  |
|  | b) | Araştırmanın Amacı | | |  |  |  |  |  |
|  | c) | Araştırma Anketi | | |  |  |  |  |  |
|  | d) | İstenen Tetkik Formu / Test Formu | | | |  |  |  |  |
|  | e) | Araştırmanın Materyal Metod | | |  |  |  |  |  |
|  | f) | Araştırmacıların cv'si | | |  |  |  |  |  |
| 5) | Bilgilendirilmiş Gönüllü Olur Formu Örneği | | | | |  |  |  |  |
| 6) | Sözleşmeler | | |  |  |  |  |  |  |
| 7) | Araştırma Broşürü | | |  |  |  |  |  |  |
| 8) | Araştırma Bütçesi | | |  |  |  |  |  |  |
| 9) | Sigorta (İlaç Çalışmalarında) | | | |  |  |  |  |  |
| 10) | Literatür | |  |  |  |  |  |  |  |
| Düşünce ve Yorumlar | | | | | | | | | |


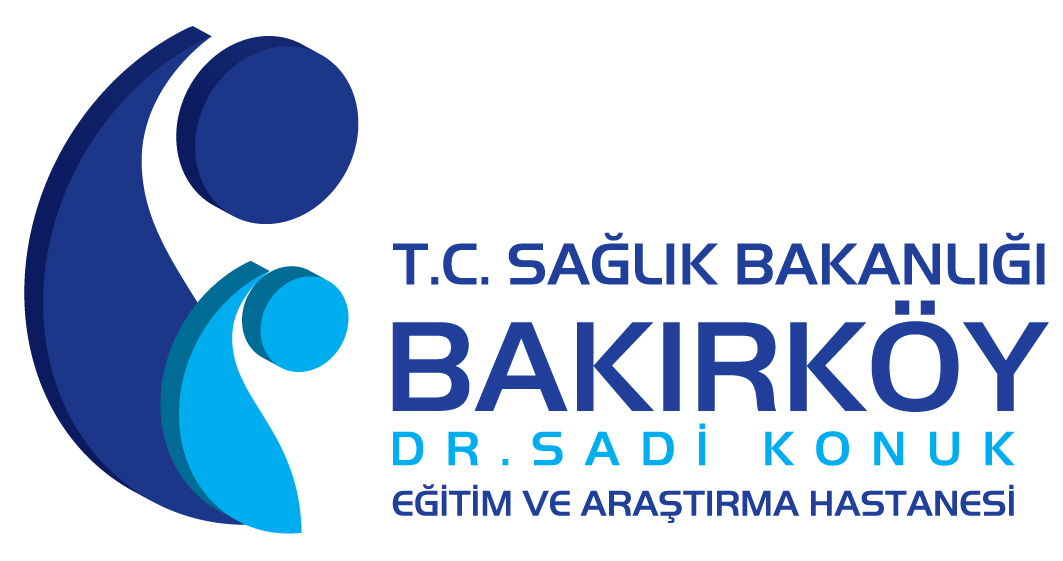

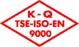


Tarih: 30.05.2015

**T.C.**

**SAĞLIK BAKANLIĞI**

**BAKIRKÖY DR. SADİ KONUK EĞİTİM VE ARAŞTIRMA HASTANESİ**

**KLİNİK ARAŞTIRMALAR ETİK KURUL BAŞKANLIĞINA,**

Süleymaniye Kadın Doğum ve Çocuk Hastalıkları Eğitim ve Araştırma Hastanesi Anestezi Bölümünde gerçekleştirilmesi düşünülen “*Fetal distres gelişen gebelerde, anestezi tekniğinin maternal ve neonatal morbidite üzerine etkinliğinin araştırılması*” başlıklı klinik ilaç dışı araştırmasının Klinik Araştırmalar Etik Kurulunuz tarafından değerlendirilmesi için gereğini saygılarımla arz ederim.

Sorumlu Araştırmacı

Ad, Soyad, imza:

Dr Saadet İpek Edipoğlu

| **T.C**  **SAĞLIK BAKANLIĞI**  **BAKIRKÖY DR. SADİ KONUK EĞİTİM VE ARAŞTIRMA HASTANESİ**  **KLİNİK ARAŞTIRMALAR ETİK KURULU** | |
| --- | --- |
| **ÖN BİLGİ FORMU** | |
| | **TANIMLAYICI BİLGİLER** | | | | | | | --- | --- | --- | --- | --- | --- | | **Sorumlu Araştırıcı** | | | Dr. Saadet İpek Edipoğlu | | | | **Kurumu** | | | Süleymaniye Kadın Hastalıkları ve Çocuk Hastalıkları Hastanesi | | | | **İletişim Bilgileri** | | **Tel** | 05535915257 | | | | **E-Posta** | ipekedipoglu@gmail.com | | | | **Yardımcı Araştırıcılar** | | | Yoktur. | | | | **İletişim Bilgileri** | | **Tel** |  | | | | **E-Posta** |  | | | | **Araştırma Adı** | | | Fetal distres gelişen gebelerde, anestezi tekniğinin maternal ve neonatal morbidite üzerine etkinliğinin araştırılması | | | | **Araştırmanın Türü** | | | Prospektif | | | | **Araştırmanın Süresi** | | | 3 ay | | | |  | | | | | | | **EK BİLGİLER** | | |  | | | | | **Bu bölüm Klinik Araştırmalar Etik Kurulu Sekreterliği tarafından doldurulacaktır** | | | | | | | **DOSYA ALINDI**  **TARİHİ** | **PROTOKOL NUMARASI** | | | **KURUL TOPLANTI TARİHİ** | **KARAR NO** | |  |  | | |  |  | |  |

| 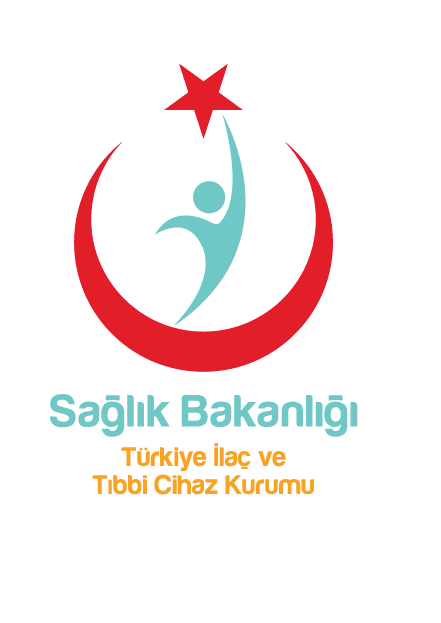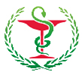 | **İLAÇ, BİYOLOJİK VE TIBBİ ÜRÜNLER BAŞKAN YARDIMCILIĞI**  **KLİNİK İLAÇ ARAŞTIRMALARI DAİRE BAŞKANLIĞI**  ÖZGEÇMİŞ FORMU | **Doküman Adı:** KADB-F12-R.00 |
| --- | --- | --- |
|  |  | **Yayın Tarihi:** 09.04.2012 |
|  |  | **Sayfa No:** 5/19 |
|  |  | **Onaylayan:** DB |

1. **KİŞİSEL BİLGİLER**

|  | **Adı soyadı:** Saadet İpek Edipoğlu |
| --- | --- |
|  | **Doğum tarihi ve yeri:** Ankara/1981 |
|  | **Yabancı dil bilgisi:** İngilizce |
|  | **Görev yeri:** Süleymaniye Kadın Doğum ve Çocuk Hastalıkları Eğitim ve Araştırma Hastanesi |
|  | **İletişim bilgileri** *(e-posta adresi / telefon)***:** ipekedipoglu@gmail.com /05535915257 |

1. **EĞİTİM BİLGİLERİ**

| **B.1** | **Mezun olduğu üniversite / fakülteyi** **lütfen belirtiniz**: İstanbul Üniversitesi Cerrahpaşa Tıp Fakültesi |
| --- | --- |
| **B.2** | **Mezuniyet tarihini lütfen belirtiniz** *(yıl olarak)*: 2005 |
| **B.3** | **Varsa, akademik ünvanları lütfen belirtiniz:** |

1. **İŞ TECRÜBESİNE AİT BİLGİLER**

| **C.1** | **Bugüne kadar çalıştığı kurum / kuruluşları lütfen belirtiniz:**  2007-2012 İstanbul Üniversitesi İstanbul Tıp Fakültesi  2012-2013 İstanbul Üniversitesi Cerrahpaşa Tıp Fakültesi  2013-2015 Süleymaniye Kadın Doğum ve Çocuk Hastalıkları Eğitim ve Araştırma Hastanesi |
| --- | --- |

1. **KLİNİK ARAŞTIRMALARLA İLGİLİ GENEL BİLGİLER**

| **D.1** | **İyi Klinik Uygulamalar (İKU) konusunda eğitim alınmışsa lütfen tarihi ve alınan kurum / kuruluşun adı ile belirtini***z*: |
| --- | --- |
| **D.2** | **Varsa, araştırmacı olarak katılınan klinik araştırmaları lütfen belirtiniz:** İstanbul Tıp Fakültesi’nde gerçekleştirilen hastane içi kardiyopulmoner resüsitasyonların değerlendirilmesi isimli çalışma (2012) |
| **D.3** | **Varsa, izleyici (monitör) olarak katılınan klinik araştırmaları lütfen belirtiniz:** |
| **D.4** | **Varsa, saha görevlisi olarak katılınan klinik araştırmaları lütfen belirtiniz:** |
| **D.5** | **Varsa, araştırma eczacısı olarak katılınan klinik araştırmaları lütfen belirtiniz:** |

1. **ÖZGEÇMİŞ SAHİBİNİN İMZASI**

| **E.2** | **Özgeçmiş Sahibi** |
| --- | --- |
| **E.2.1** | El yazısıyla adı soyadı: |
| **E.2.2** | Tarih (gün/ay/yıl olarak): 30.05.2015 |
| **E.2.3** | İmza: |

| 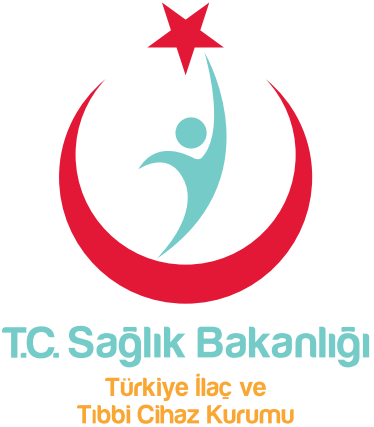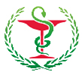 | **KLİNİK ARAŞTIRMALAR**  **BAŞVURU FORMU**  **(UZMANLIK TEZİ VEYA**  **AKADEMİK AMAÇLI YAPILACAK)** | **Doküman Adı:** KADB-F.03-R.03 |
| --- | --- | --- |
|  |  | **Yayın Tarihi:** 04.07.2014 |
|  |  | **Sayfa No:** 6/19 |
|  |  | **Onaylayan:** Daire Başkanı |

1. **BAŞVURUNUN YAPILDIĞI YER**

*Türkiye İlaç ve Tıbbi Cihaz Kurumu ve Klinik Araştırmalar Etik Kurulun*a yapılacak olan başvurular için aynı form kullanılmalı ve ilgili kutu işaretlenmelidir.

| **A.1.** | **Türkiye İlaç ve Tıbbi Cihaz Kurumu** |  |
| --- | --- | --- |
| **A.2.** | **Klinik Araştırmalar Etik Kurulu** |  |

1. **ARAŞTIRMA**

| **B.1.** | **Araştırmanın açık adı:** Fetal distres gelişen gebelerde, anestezi tekniğinin maternal ve neonatal morbidite üzerine etkinliğinin araştırılması | | | |
| --- | --- | --- | --- | --- |
|  | | | | |
| **B.2.** | **Varsa, protokol numarası:** | | | |
|  | | | | |
| **B.3.** | **Araştırma pediatrik popülasyon üzerinde yürütülecek mi?** | Evet | Hayır | |
|  |  |  |  | |
| **B.4.** | **Araştırma amacıyla kullanılan etkin maddenin adı:** | | | |
|  |  | | | |
| **B.4.1.** | **Varsa, karşılaştırma amacıyla kullanılan etkin maddenin adı:** | | | |
|  |  | | | |
| **B.4.2.** | **Araştırma ürünü ruhsatlı/izinli ise müstahzar adı:** | | | |
|  | | | | |
| **B.5.** | **Araştırmanın statüsü** (*aşağıdaki kutucuklardan uygun olanı işaretleyiniz*) | | | |
| **B.5.1.** | Yüksek lisans tezi | | |  |
| **B.5.2.** | Doktora tezi | | |  |
| **B.5.3.** | Uzmanlık tezi | | |  |
| **B.5.4.** | Bireysel araştırma projesi | | |  |
| **B.5.5.** | Diğer ise lütfen belirtiniz: | | | |

1. **DESTEKLEYİCİ**

| **C.1.** | **Araştırmanın destekleyicisi var mı?** | Evet | Hayır | |
| --- | --- | --- | --- | --- |
| **C.1.1.** | C.1’e cevabınız evet ise lütfen aşağıdaki uygun kutucuğu işaretleyiniz. | | | |
| **C.1.1.1.** | Üniversite | | |  |
| **C.1.1.2.** | Eğitim ve Araştırma Hastanesi | | |  |
| **C.1.1.3.** | TÜBİTAK (*Türkiye Bilimsel ve Teknolojik Araştırma Kurumu*) | | |  |
| **C.1.1.4.** | Uluslararası ise lütfen belirtiniz: | | | |
| **C.1.1.5.** | Diğer ise lütfen belirtiniz: | | | |

1. **ARAŞTIRMAYA İLİŞKİN GENEL BİLGİLER**

Bu bölüm, araştırmanın amacı, kapsamı ve tasarımına ilişkin bilgilerin sağlanması için kullanılmalıdır.

| **D.1.** | **Araştırılan tıbbi durum veya hastalık** | | | | | | | | |
| --- | --- | --- | --- | --- | --- | --- | --- | --- | --- |
| **D.1.1.** | Araştırılan tıbbi durum veya hastalıkları lütfen belirtiniz (*serbest metin olarak belirtiniz*): | | | | | | | | |
| **D.1.1.1.** | Tedavi alanını (onkoloji, hematoloji gibi) lütfen belirtiniz: Hastalara çalışma amaçlı tedavi verilmeyecektir | | | | | | | | |
| **D.1.2.** | Araştırılan durumlardan herhangi biri nadir bir hastalık mı? | | | Evet | | | Hayır | | |
| **D.1.2.1.** | D.1.2’ye cevabınız evet ise lütfen belirtiniz: | | | | | | | | |
|  | | | | | | | | | |
| **D.2.** | **Araştırmanın amacı** | | | | | | | | |
| **D.2.1.** | Primer amaç: Fetal distres nedeniyle sezeryan yapılmış gebelerde, anestezi tekniğinin neonatal morbidite üzerine etkinliğinin retrospektif şekilde araştırılmasıdır. | | | | | | | | |
|  | | | | | | | | | |
| **D.3.** | **Gönüllülerin araştırmaya dâhil edilme kriterleri** *(lütfen maddeler halinde sıralayınız)*: Çalışmamız retrospektif olduğu için gönüllü yoktur. Araştırmaya dâhil edilme kriterleri  -18-50 yaş arası hastalar dâhil edilecektir  -BMI <40’ın üzerinde olanlar,  -Nörolojik bozukluğu bulunmayan hastalar  -Kanama bozukluğu olmayan | | | | | | | | |
|  | | | | | | | | | |
| **D.4.** | **Gönüllülerin araştırmaya dâhil edilmeme kriterleri** *(lütfen maddeler halinde sıralayınız)*:  -18 yaş altı hastalar  -BMI > 40’ın üzerinde olanlar,  -Nörolojik bozukluğu bulunan hastalar  -Kanama bozukluğu olan hastalar | | | | | | | | |
|  | | | | | | | | | |
| **D.5.** | **Araştırmanın kapsamı** (*Lütfen uygun olan kutuları işaretleyiniz*) | | | | | | | | |
| **D.5.1.** | Teşhis | | | | | |  | | |
| **D.5.2.** | Tedavi | | | | | |  | | |
| **D.5.3.** | Profilaksi | | | | | |  | | |
| **D.5.4.** | Güvenilirlik | | | | | |  | | |
| **D.5.5.** | Etkililik | | | | | |  | | |
| **D.5.6.** | Farmakokinetik | | | | | |  | | |
| **D.5.7.** | Farmakodinamik | | | | | |  | | |
| **D.5.8.** | Farmakogenetik | | | | | |  | | |
| **D.5.9.** | Farmakoekonomik | | | | | |  | | |
| **D.5.10.** | Diğer ise, lütfen belirtiniz: | | | | | | | | |
|  | | | | | | | | | |
| **D.6.** | **Araştırmanın fazı** (*Lütfen uygun olan kutuyu işaretleyiniz*) | | | | | | | | |
| **D.6.1.** | Faz I | | | | | | |  | |
| **D.6.2.** | Faz II | | | | | | |  | |
| **D.6.3.** | Faz III | | | | | | |  | |
| **D.6.4.** | Faz IV | | | | | | |  | |
|  | | | | | | | | | |
| **D.7.** | **Araştırmanın tasarımı** (*Lütfen uygun olan kutu/kutuları işaretleyiniz*) | | | | | | | | |
| **D.7.1.** | Kontrollü | Evet | | | | Hayır | | | |
| **D.7.1.1.** | Kontrollü ise, lütfen karşılaştırma ürününü belirtiniz: | | | | | | | | |
| **D.7.2.** | Randomize | Evet | | | | Hayır | | | |
| **D.7.3.** | Açık etiketli | Evet | | | | Hayır | | | |
| **D.7.4.** | Tek kör | Evet | | | | Hayır | | | |
| **D.7.5.** | Çift kör | Evet | | | | Hayır | | | |
| **D.7.6.** | Çift sağır (Double-dummy) | Evet | | | | Hayır | | | |
| **D.7.7.** | Paralel grup | Evet | | | | Hayır | | | |
| **D.7.8.** | Çapraz (cross-over) | Evet | | | | Hayır | | | |
| **D.7.9.** | Diğer ise lütfen belirtiniz: | | | | | | | | |
|  | | | | | | | | | |
| **D.8.** | **Araştırma merkezi** | | | | | | | | |
| **D.8.1.** | Tek bir merkez var | Evet | | | | Hayır | | | |
| **D.8.2.** | Birden çok merkez var | Evet | | | | Hayır | | | |
| **D.8.2.1.** | Ülkemizde öngörülen merkez sayısı ve isimlerini lütfen belirtiniz: | | | | | | | | |
| **D.8.3.** | Bu araştırma başka ülkelerde de yürütülüyor mu? | Evet | | | | Hayır | | | |
| **D.8.3.1.** | D.8.3’e cevabınız evet ise başka ülkelerde öngörülen merkez sayısını ve ülkeleri lütfen belirtiniz: | | | | | | | | |
|  | | | | | | | | | |
| **D.9.** | **Bu araştırmada bağımsız bir veri izleme komitesi var mı?** | Evet | | | | Hayır | | | |
| **D.9.1.** | D.9’a cevabınız evet ise komitenin yapısına ve iletişim bilgilerine ait bilgileri belirtiniz: | | | | | | | | |
|  | | | | | | | | | |
| **D.10.** | **Araştırma süresi** | | | | | | | | |
| **D.10.1.** | Araştırmanın tahminen ne kadar süreceği[[1]](#footnote-2) (*gün, ay ve yıl olarak*): 3 ay | | | | | | | | |
| **D.10.1.1.** | Araştırmanın tahminen ülkemizde ne kadar süreceğini lütfen belirtiniz: | | 01 | | 11 | | | | 2015 |
| **D.10.1.2.** | Varsa, araştırmada yer alan bütün ülkelerde araştırmanın tahminen ne kadar süreceğini lütfen belirtiniz: | |  | |  | | | |  |
| **D.10.2.** | Araştırmaya gönüllü almaya başlamak için önerilen tarih (*gün, ay ve yıl olarak*): | | | | | | | | |
| **D.10.2.1.** | Ülkemizdeki tarihi lütfen belirtiniz: | | 01 | | 08 | | | | 2015 |
| **D.10.2.2.** | Varsa, diğer ülkelerdeki tarihi lütfen belirtiniz: | |  | |  | | | |  |

1. **ARAŞTIRMA ÜRÜNÜNE İLİŞKİN BİLGİLER**

Araştırmanın birden çok ürün içermesi halinde ek sayfalar kullanarak belirtilmelidir. Ürünün kombinasyon ürünü olması halinde her bir etken madde için ayrı bilgi verilmelidir.

| **E.1** | **Araştırma ürünü [[2]](#footnote-3)** |
| --- | --- |
| **E.1.1** | Test edilen araştırma ürününü lütfen belirtiniz: yoktur |
| **E.1.2** | Varsa, karşılaştırma amacıyla kullanılan araştırma ürününü lütfen belirtiniz: yoktur |

| **E.2** | **Araştırma ürününün statüsü^3^** | | | |
| --- | --- | --- | --- | --- |
| **E.2.1** | Araştırmada kullanılacak olan araştırma ürünü ruhsatlı mı? | Evet | Hayır | |
| **E.2.2** | E.2.1’e cevabınız evet ise, lütfen araştırmada kullanılacak ürününün ruhsatlı olduğu ülkeleri belirtiniz: | | | |
| **E.2.3** | Araştırma ürününde ruhsata göre değişiklik yapılmış mı? | Evet | Hayır | |
| **E.2.3.1** | E.2.3’e cevabınız evet ise lütfen belirtiniz: | | | |
| **E.2.4** | Kullanılan araştırma ürünü, ülkemizde, Avrupa Birliği üye ülkelerinde veya Amerika Birleşik Devletleri içinde bu endikasyon için yetim ilaç olarak nitelendirilmiş mi? | Evet | Hayır | |
| **E.2.4.1** | E.2.4’e cevabınız evet ise, belge örneğini lütfen başvuru dosyasına ekleyiniz. | | | |
|  | | | | |
| **E.3** | **Araştırma ürünü ülkemizde ruhsatlı/izinli ise kullanma talimatı/kısa ürün bilgisi örneğini lütfen başvuru dosyasına ekleyiniz.** | | | |
|  | | | | |
| **E.4** | **Kullanılan araştırma ürünü, daha önce destekleyicinin ülkemizde, Avrupa Birliği üye ülkelerinde veya Amerika Birleşik Devletleri içinde gerçekleştirdiği bir klinik araştırmada kullanılması için onay almış mı?** | Evet | | Hayır |
| **E.4.1** | E.4’e cevabınız evet ise ülkeleri belirtiniz: | | | |
|  | | | | |
| **E.5** | **Araştırma ürünü ilk kez insanlarda uygulanan bir klinik araştırmada mı kullanılacak?** | Evet | Hayır | |
| **E.5.1** | E.5’e cevabınız evet ise belirlenen risk faktörleri var mı? | Evet | Hayır | |
| **E.5.2** | E.5.1’e cevabınız evet ise lütfen belirtiniz: | | | |

| **E.6** | **Araştırma ürününün farmasötik formu, uygulama yolu ve etki mekanizması** | | |
| --- | --- | --- | --- |
| **E.6.1** | Araştırma ürününün farmasötik formunu belirtiniz *(lütfen standart ifadeler kullanınız):* | | |
| **E.6.2** | Uygulama yolunu belirtiniz *(lütfen standart ifadeler kullanınız):* | | |
| **E.6.3** | Etki mekanizmasını belirtiniz *(lütfen serbest metin olarak belirtiniz):* | | |
|  | | | |
| **E.7** | **Araştırma ürünü aşağıdaki etken maddelerden hangisini içeriyorsa lütfen uygun kutucuğu işaretleyiniz.** | | |
| **E.7.1** | Kimyasal kökenli mi? | Evet | Hayır |
| **E.7.2** | Biyolojik kökenli mi? | Evet | Hayır |
| **E.7.3** | Somatik hücre tedavisi tıbbi ürünü mü? | Evet | Hayır |
| **E.7.4** | Gen tedavisi tıbbi ürünü mü? | Evet | Hayır |
| **E.7.5** | Radyofarmasötik tıbbi ürün mü? | Evet | Hayır |
| **E.7.6** | İmmünolojik tıbbi ürün mü (aşı, alerjen veya immün serum gibi)? | Evet | Hayır |
| **E.7.7** | Plazma türevli tıbbi ürün mü? | Evet | Hayır |
| **E.7.8** | Bitkisel tıbbi ürün mü? | Evet | Hayır |
| **E.7.9** | Homeopatik tıbbi ürün mü? | Evet | Hayır |
| **E.7.10** | Genetiği değiştirilmiş organizma içeren tıbbi ürün mü? | Evet | Hayır |
| **E.7.10.1** | E.7.10 için cevabınız evet ise, ruhsat verilmiş mi? | Evet | Hayır |
| **E.7.10.2** | E.7.10.1 için cevabınız hayır ise, ruhsat başvurusu yapılmış mı? | Evet | Hayır |
| **E.7.11** | Başka bir tür tıbbi ürün mü? | Evet | Hayır |
| **E.7.11.1** | E.7.11’e cevabınız evet ise, lütfen belirtiniz: | | |

| **E.8** | **Aşılar dahil, araştırılan biyolojik tıbbi ürünlerin türü** | | |
| --- | --- | --- | --- |
| **E.8.1** | Ekstraktif | Evet | Hayır |
| **E.8.2** | Rekombinant | Evet | Hayır |
| **E.8.3** | Aşı | Evet | Hayır |
| **E.8.4** | Genetik modifiye organizma (GMO) | Evet | Hayır |
| **E.8.5** | Plazma türevli ürünler | Evet | Hayır |
| **E.8.6** | Diğer ise, lütfen belirtiniz: | | |

| **E.9** | **Araştırılan somatik hücre tedavisi tıbbi ürünlerde hücrelerin kökeni (genetiği değişikliğe uğratılmamış)** | | |
| --- | --- | --- | --- |
| **E.9.1** | Otolog | Evet | Hayır |
| **E.9.2** | Allojenik | Evet | Hayır |
| **E.9.3** | Ksenojenik | Evet | Hayır |
| **E.9.3.1** | E.9.3’e cevabınız evet ise, lütfen köken türlerini belirtiniz: | | |
| **E.9.4** | Diğer ise, lütfen belirtiniz: | | |
|  | | | |
| **E.10** | **Araştırılan somatik hücre tedavisi tıbbi ürünlerde hücre türleri (genetiği değişikliğe uğratılmamış)** | | |
| **E.10.1** | Kök hücreler | Evet | Hayır |
| **E.10.2** | Ayrıştırılmış hücreler | Evet | Hayır |
| **E.10.2.1** | E.10.2’ye cevabınız evet ise, lütfen türünü belirtiniz (keratinositler, fibroblastlar, kondrositler gibi): | | |
| **E.10.3** | Diğer ise, lütfen belirtiniz: | | |
|  | | | |
| **E.11** | **Araştırılan gen tedavisi tıbbi ürünlerinde ilgilenilen genler** | | |
| **E.11.1** | In vivo gen tedavisi | Evet | Hayır |
| **E.11.2** | Ex vivo gen tedavisi | Evet | Hayır |
|  | | | |
| **E.12** | **Araştırılan gen tedavisi tıbbi ürünlerinlerinde gen transfer ürünün türü** | | |
| **E.12.1** | Nükleik asit (örneğin plasmid) | Evet | Hayır |
| **E.12.1.1** | Salt (Naked) | Evet | Hayır |
| **E.12.1.2** | Kompleks | Evet | Hayır |
| **E.12.2** | Viral vektör | Evet | Hayır |
| **E.12.2.1** | E.12.2’ye cevabınız evet ise, lütfen türünü belirtiniz: (adenovirüs, retrovirüs gibi) | | |
| **E.12.3** | Diğer ise, lütfen belirtiniz: | | |
| **E.12.4** | Genetiği değiştirilmiş hücreler | Evet | Hayır |
| **E.12.5** | Otolog | Evet | Hayır |
| **E.12.6** | Allojenik | Evet | Hayır |
| **E.12.7** | Ksenojenik | Evet | Hayır |
| **E.12.7.1** | E.12.7’ye cevabınız evet ise, lütfen köken türlerini belirtiniz: | | |
| **E.12.8** | Diğer hücre türleri (hematopoetik kök hücreler gibi) ise, lütfen belirtiniz: | | |
|  | | | |
| **E.13** | **Mevcutsa, araştırılan gen tedavisi tıbbi ürününe ilişkin yeni yönler hakkında yorumları lütfen belirtiniz** *(lütfen* *serbest metin olarak belirtiniz*): | | |

| **E.14** | **Plasebo kullanılıyor mu?** | Evet | Hayır |
| --- | --- | --- | --- |

1. ARAŞTIRMADAKİ GÖNÜLLÜ POPÜLASYONU

| **F.1.** | **Yaş aralığı** *(Araştırmanın tamamı için her yaş aralığında planlanan tahmini gönüllü sayısını belirtiniz)* | | | | |
| --- | --- | --- | --- | --- | --- |
| **F.1.1.** | 18 yaş altı | | Evet | Hayır | |
| **F.1.1.1** | F.1.1’e cevabınız evet ise lütfen yaş aralığını ve gönüllü sayısını belirtiniz: | | | | |
| **F.1.2.** | 18 yaş üstü | | Evet | Hayır | |
| **F.1.2.1.** | F.1.2’ye cevabınız evet ise lütfen yaş aralığını ve gönüllü sayısını belirtiniz: 18-50 yaş arası | | | | |
|  | | | | | |
| **F.2.** | **Cinsiyet** | | | | |
| **F.2.1.** | Kadın | | | |  |
| **F.2.2.** | Erkek | | | |  |
|  | | | | |  |
| **F.3.** | **Araştırmadaki gönüllü grubu** | | | | |
| **E.3.1.** | Sağlıklı gönüllüler | Evet | | Hayır | |
| **F.3.2.** | Hastalar | Evet | | Hayır | |
| **F.3.3.** | Özel hassas popülâsyonlar | Evet | | Hayır | |
| **F.3.3.1.** | Doğum kontrol yöntemi kullanmayan ve çocuk doğurma potansiyeli olan kadınlar | Evet | | Hayır | |
| **F.3.3.2.** | Doğum kontrol yöntemi kullanan ve çocuk doğurma potansiyeli olan kadınlar | Evet | | Hayır | |
| **F.3.3.3.** | Gebe kadınlar | Evet | | Hayır | |
| **F.3.3.4.** | Emziren kadınlar | Evet | | Hayır | |
| **F.3.3.5.** | Acil vakalar | Evet | | Hayır | |
| **F.3.3.6.** | Şahsen olur veremeyecek gönüllüler | Evet | | Hayır | |
| **F.3.3.6.1.** | F.3.3.6’ya cevabınız evet ise lütfen belirtiniz: | | | | |
| **F.3.3.7.** | Diğer ise, lütfen belirtiniz: | | | | |

1. BAŞVURUDA YER ALAN KLİNİK ARAŞTIRMA MERKEZLERİ/ARAŞTIRMACILAR

| **G.1.** | **Koordinatör** *(Çok merkezli araştırmalar için)* **ve sorumlu araştırmacı** *(Tek merkezli araştırmalar için)* |
| --- | --- |
| **G.1.1.** | Adı Soyadı: Saadet İpek Edipoğlu |
| **G.1.2.** | Unvanı: Uzman |
| **G.1.3.** | Uzmanlık alanı: Anesteziyoloji ve Reanimasyon |
| **G.1.4.** | Kurumu: Süleymaniye Kadın doğum ve Çocuk Hastalıkları Eğitim ve Araştırma Hastanesi |
| **G.1.5.** | Telefon numarası: 05535915257 |
| **G.1.6.** | E-posta adresi: ipekedipoglu@gmail.com |

| **G.2.** | **İdari Sorumlu** |
| --- | --- |
| **G.2.1.** | Adı Soyadı: Saadet İpek Edipoğlu |
| **G.2.2.** | Unvanı: Uzman |
| **G.2.3.** | Uzmanlık alanı: Anesteziyoloji ve Reanimasyon |
| **G.2.4.** | Kurumu: Süleymaniye Kadın doğum ve Çocuk Hastalıkları Eğitim ve Araştırma Hastanesi |
| **G.2.5.** | Telefon numarası: 05535915257 |

| **G.3.** | **Sorumlu araştırmacı** *(Çok merkezli araştırmalar için gerektiğinde bu bölümü lütfen tekrarlayınız)* |
| --- | --- |
| **G.3.1.** | Adı Soyadı: |
| **G.3.2.** | Unvanı : |
| **G.3.3.** | Uzmanlık alanı: |
| **G.3.4.** | Kurumu: |
| **G.3.5.** | Telefon numarası: |
| **G.3.6.** | E-posta adresi: |
|  | |
| **G.4.** | **Yardımcı araştırmacı** *(Gerektiğinde bu bölümü lütfen tekrar ediniz)* |
| **G.4.1.** | Adı Soyadı: |
| **G.4.2.** | Unvanı: |
| **G.4.3.** | Uzmanlık alanı: |
| **G.4.4.** | Kurumu: |
| **G.4.5.** | Telefon numarası: |
| **G.4.6.** | E-posta adresi: |
|  | |
| **G.5.** | **Varsa izleyici (monitör) bilgileri** *(Birden çok kişi olması halinde bu bölümü lütfen tekrarlayınız)* |
| **G.5.1.** | İzleyicinin adı soyadı: |
| **G.5.2.** | Bağlı bulunduğu Kurum/Kuruluşun adı: |
| **G.5.3.** | Telefon numarası: |
| **G.5.4.** | E-posta adresi: |

| **G.6.** | **Varsa araştırma eczacısı** *(Gerektiğinde bu bölümü lütfen tekrar ediniz)* |
| --- | --- |
| **G.6.1.** | Adı Soyadı: |
| **G.6.2.** | Kurumu: |
| **G.6.3.** | Telefon numarası: |
| **G.6.4.** | E-posta adresi: |

| **G.7.** | **Araştırmanın gerçekleştirilmesinde kullanılacak olan merkezi teknik tesisler, temel değerlendirme kriterlerinin ölçümü veya değerlendirilmesinin merkezileştirildiği laboratuar veya diğer teknik tesisleri lütfen belirtiniz** *(Birden çok organizasyon olması halinde lütfen gerektiği kadar tekrarlayınız. Bu amaçla ek sayfalar kullanabilirsiniz)***:** |
| --- | --- |
| **G.7.1.** | Organizasyon: |
| **G.7.2.** | Temasa geçilecek kişinin adı soyadı: |
| **G.7.3.** | Adresi: |
| **G.7.4.** | Telefon numarası: |
| **G.7.5.** | Dışarıya verilen görevler: yoktur |

1. **ETİK KURUL BİLGİLERİ**

***Bu bölüm, Türkiye İlaç ve Tıbbi Cihaz Kurumu’na başvuru yapılırken doldurulmalıdır.***

| **H.1.** | **Araştırma onayı için etik kurul başvurusu yapıldı mı?** | Evet | Hayır |
| --- | --- | --- | --- |
| **H.1.1.** | H.1’e cevabınız evet ise; | | |
| **H.1.1.1.** | Klinik Araştırmalar Etik kurulunun adı: | | |
| **H.1.1.2.** | Başvuru tarihi: | | |
| **H.2.** | **Araştırmaya ait etik kurul onayı var mı?** | Evet | Hayır |
| **H.2.1.** | H.2’ye cevabınız evet ise etik kurul kararının aslı veya aslı gibidir onaylı örneğini başvuru dosyasına ekleyiniz.  *Etik kurul karar formunun aslı gibidir onayı etik kurul başkanı veya etik kurul sekretaryası tarafından yapılmalıdır.* | | |

1. **İLGİLİ BELGELER**

Bu bölümde belirtilen belgeler sırası ile başvuru dosyasına eklenmelidir.

| **İ.1.** | **Varsa, daha önce ret edilen etik kurul kararının aslı veya aslı gibidir onaylı örneği sunulmalıdır.** |
| --- | --- |
| **İ.2.** | **Anabilim dalı başkanı veya eğitim sorumlusu tarafından onaylanan belge***  *Araştırmanın uzmanlık tezi veya akademik amaçlı olduğuna dair Anabilim Dalı Başkanı veya Eğitim Sorumlusu tarafından onaylanan ıslak imzalı belge sunulmalıdır. |
| **İ.3.** | **Araştırma protokolü***  Varsa, Tarihi:      Versiyon numarası:  *****Protokol, çok merkezli araştırmalar için koordinatör, tek merkezli araştırmalar için sorumlu araştırmacı tarafından imzalanmalıdır. |
| **İ.3.1.** | **Varsa, Türkçe protokol özeti** |
| **İ.4.** | **Araştırma akış şeması** |
| **İ.5.** | **Bilgilendirilmiş Gönüllü Olur Formu (BGOF)***  Tarihi:       Versiyon numarası:  *BGOF’nin [www.titck.gov.tr](http://www.titck.gov.tr/) adresinde yer alan asgari bilgilendirilmiş gönüllü olur formu örneği doğrultusunda hazırlanması ve başvuru dosyasına eklenmesi gerekmektedir. İngilizce dışındaki dillerde olan BGOF’lerin orijinali ve yeminli tercüman tarafından onaylı tercümesinin ilave edilmesi gerekmektedir. |
| **İ.6.** | **Olgu Rapor Formu (ORF) ***  Tarihi:       Versiyon numarası:  *****“İyi Klinik Uygulamaları Kılavuzu” doğrultusunda hazırlanmış “Araştırmadaki her bir gönüllüye ait verilerin ve diğer bilgilerin araştırma protokolünde tanımlandığı şekilde kaydının yapılması için hazırlanan basılı, optik veya elektronik belge” |
| **İ.7.** | **Araştırma broşürü** *(Varsa)*  Tarihi:       Versiyon numarası: |
| **İ.8.** | **Ülkemizde ruhsatlı/izinli bir araştırma ürünü ile çalışma yapılıyorsa araştırma ürününe ait kısa ürün bilgisi (KÜB)-kullanma talimatı (KT) örneği** |
| **İ.9.** | **Sigorta*** (Faz IV dışındaki araştırmalar için)  * Sigorta, [www.titck.gov.tr](http://www.titck.gov.tr/) adresinde yer alan “Klinik Araştırmalarda Yapılacak Olan Sigorta Teminatına İlişkin Kılavuz” doğrultusunda hazırlanmalıdır. |
| **İ.10.** | **Araştırma bütçesi***  * Bütçe formu, [www.titck.gov.tr](http://www.titck.gov.tr/) adresinde yer alan güncel formatta yetkili kişiler (çok merkezli araştırmalar için koordinatör, tek merkezli araştırmalar için sorumlu araştırmacı*)* tarafından ıslak imzalı olmalıdır. |
| **İ.11.** | **Koordinatörün/İdari Sorumlunun (tek merkezli araştırmalarda sorumlu araştırmacının) ve varsa araştırma eczacısının özgeçmişi***  *Özgeçmiş formu, [www.titck.gov.tr](http://www.titck.gov.tr/) adresinde yer alan güncel formatta adı soyadı ve unvanı el yazısı ile yazılmış, tarihli ve ıslak imzalı olmalıdır. |
| **İ.12.** | **Gerekli ise, Biyolojik Materyal Transfer Formu örneği (BMTF)*:**  *BMTF, [www.titck.gov.tr](http://www.titck.gov.tr/) adresinde yer alan güncel formatta ve ıslak imzalı olmalıdır. |
| **İ.13.** | **Varsa yetkilendirme belgeleri** |
| **İ.14.** | **Varsa gönüllü bilgilendirme metinleri** |
| **İ.15.** | **Varsa anketler** |
| **İ.16.** | **Varsa ilanlar** |
| **İ.17.** | **Varsa hasta kartı/günlüğü**  Versiyon numarası:       Tarihi: |
| **İ.18.** | **Araştırmaya ilişkin destekleyici belge / literatürler** |
| **İ.19.** | **Başvuru dosyası ekinde yer alan belgelerin yer aldığı CD***  *Belgeler pdf formatında olmalıdır (imzalı olması gerekli olan belgeler imzalanarak eklenmelidir). |

**J**. BAŞVURU SAHİBİNİN İMZASI

| **J.1.** | **İşbu başvuru formuyla, şahsım / başvuru sahibi adına** *(lütfen geçerli olmayan ifadelerin üzerini yanına tarih ve paraf atarak çiziniz)* |
| --- | --- |
|  | - Başvuruda sağlanan bilgilerin doğru olduğunu, - Araştırmanın protokole, ilgili mevzuata, güncel Helsinki Bildirgesi ve iyi klinik uygulamaları ilkelerine uygun olarak gerçekleştirileceğini, - Araştırma ürününün İyi İmalat Uygulamaları (İİU) kılavuzuna uygun olarak üretildiğini, - Araştırma ürününe ait Türkçe etiket örneğinin İyi İmalat Uygulamaları (İİU) kılavuzuna uygun olarak hazırlandığını, - Araştırma ekibini (laborutuvar ekibi, araştırma hemşiresi, araştırma eczacısı vb. dahil) araştırma hakkında bilgilendirdiğimi, - Önerilen klinik araştırmanın gerçekleştirilebilir nitelikte olduğunu, - Araştırma başvurusunun ilgili Yönetmelik kapsamında kurulan etik kurullardan, aynı anda birden fazlasına yapılmadığını, - Ciddi advers olaylara/reaksiyonlara ilişkin raporları ve güvenililik raporlarını sunacağımı, - Araştırmanın, kişisel verilerin gizliliğine riayet edilmek kaydıyla kamuya açık bir veri tabanına kaydedildiğini, - Araştırma bütün ülkelerde/ülkemizde sona erdikten sonra, 1 (bir) yıllık azami süre içerisinde nihai raporun bir kopyasını ilgili birime sunacağımı taahhüt ederim. |
| **J.2.** | **Başvuru Sahibi** |
| **J.2.1.** | El yazısıyla adı soyadı: |
| **J.2.2.** | Tarih (gün/ay/yıl olarak): 30.05.2015 |
| **J.2.3.** | İmza: |

ARAŞTIRMA PROTOKOLÜ

ÇALIŞMANIN BAŞLIĞI

Fetal distres gelişen gebelerde, anestezi tekniğinin maternal ve neonatal morbidite üzerine etkinliğinin retrospektif araştırılması.

KONU

Çalışmamızın amacı fetal distres nedeniyle sezeryan yapılmış gebelerde, anestezi tekniğinin neonatal morbidite üzerine etkinliğinin retrospektif şekilde araştırılmasıdır. Sezeryan operasyonları doğru endikasyonla uygulandığı zaman anne ve çocuk sağlığını olumlu yönde etki eden ve zorunlu olarak uygulanan yöntemlerdir. Annenin ve fetusun mortalite ve morbiditesini azaltmak için her yıl yüzlerce çalışma yapılmaktadır. Anestezi tekniği olarak hastalara genel ya da rejyonel uygulanmaktadır. Rejyonel ve genel anestezi teknikleri ile ilgili olarak birbilerine üstünlükleri ile ilgili tartışmalar süregelmektedir.

AMAÇ

Çalışmamızın fetal distresi nedeniyle sezeryan yapılmış gebelerde, anestezi tekniğinin neonatal morbidite üzerine etkinliğinin retrospektif şekilde araştırılmasıdır. Bizim hedefimiz rutin olarak uygulanan genel ve rejyonel anestezi tekniklerinden bir tanesinin diğerine üstünlüğü olup olmadığına bir katkı yapmaktır

**ENDIKASYON YADA KOMPILIKASYONLAR**

Çalışmamız retrospektif olarak veri tarama olduğu için herhangi bir komplikasyon gelişmesi mümkün değildir. Sadece hasta dosyaları taranacaktır.

**YÖNTEM**

Retrospektif veri taramasıdır.

**MATERYAL METOD**

Hastanemizde fetal distress nedeniyle acil sezeryan geçirmiş olan hastaların dosyaları ve anestezi fişleri taranacaktır. Genel anestezi tercih edenler bir grup spinal anestezi tercih edenler diğer grup olacaktır. Haziran 2014 ile Aralık 2014 tarihleri arasında fetal distress nedeniyle acil olarak sezaryena alınmış çalışma kriterlerine uygun hastaların verileri incelenecek. Yaklaşık olarak spinal anestezi uygulanmış 50 kadın hasta ile genel anestezi uygulanmış 50 kadın hastanın verilerinin retrospektif olarak incelenmesi planlanmaktadır. Bu hastaların dosyalarından hemodinamik verileri, morbidite ve mortaliteleri karşılaştırılacaktır. Rutin olarak, bebeğin çıkışını takiben değerlendirilen, bütün yeni doğanların APGAR skorları, dosyalarından toplanarak kaydedilecek ve eğer endikasyon olmuş ve alınmışsa dosyalarında mevcut olan umblikal kord kan gazlarının sonuçları incelenecektir. Yenidoğanların ameliyat sırasında ve sonrasında gelişmiş bir komplikasyonları olduğu dosyalarında görüldüğü takdirde kayıt altına alınacaktır. Sekonder olarak epikrizlerin incelenmesi esnasında perop ya da postop dönemde annede bir komplikasyon gelişmişse de kayıt altına alınacaktır.

**KAYNAKLAR:**

1. Afolabi BB, Lesi FE a, Merah N a. Regional versus general anaesthesia for caesarean section. Cochrane Database Syst Rev. 2006;(4):CD004350.

HASTA TAKİP FORMU (EK1)

**Fetal distres gelişen gebelerde, anestezi tekniğinin maternal ve neonatal morbidite üzerine etkinliğinin retrospektif araştırılması**

| **Maternal veriler** | | | | | | | | | | | | | | | | |
| --- | --- | --- | --- | --- | --- | --- | --- | --- | --- | --- | --- | --- | --- | --- | --- | --- |
| Hasta adı: | | | | | | Yaş: | | BMI: | | Gestasyon: | | | | |  | |
| Cerrahi süre: | | | | Grup: | | Spinalden insizyona kadar geçen süre: | | | | | | | | | | |
| Perop komplikasyon (Gebe): | | | | | | Postop komplikasyon (Gebe): | | | | | | | | | | |
| Gebe Hastane çıkış: | | | | | |  | | | | | | | | | | |
| **Hemodinamik takip (dk)** | 0 | | 5 | 10 | 15 | 20 | 25 | 30 | 35 | 40 | | 45 | 50 | 55 | 60 |  |
| **TA** |  |  | |  |  |  |  |  |  |  |  | |  |  |  |  |
| **KTA** |  |  | |  |  |  |  |  |  |  |  | |  |  |  |  |
|  | | | | | | | | | | | | | | | | |
| **Neonatal Veriler** | | | | | | | | | | | | | | | | |
| Perop komplikasyon (Yenidoğan): | | | | | | | | Postop komplikasyon (Yenidoğan): | | | | | | | | |
| Bebek Hastane Çıkış: | | | | | | | |  | | | | | | | | |
| **Umblikal kan gazı** | pH: | | | pO2: | | pCO2: | | BE: | | Na: | | | K: | | Glu: | Ca: |
|  | 1dk | | | 2dk | | 3dk | | 4dk | | 5dk | | |  | |  |  |
| **APGAR skoru** |  | | |  | |  | |  | |  | | |  | |  |  |
| Ek: | | | | | | | | | | | | | | | | |

| 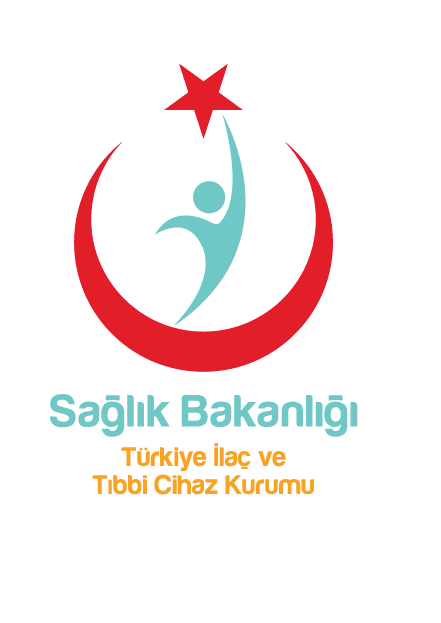 | **İLAÇ, BİYOLOJİK VE TIBBİ ÜRÜNLER BAŞKAN YARDIMCILIĞI**  **KLİNİK İLAÇ ARAŞTIRMALARI DAİRE BAŞKANLIĞI**  ARAŞTIRMA BÜTÇE FORMU | **Doküman Adı:** KADB-F16-R.00 |
| --- | --- | --- |
|  |  | **Yayın Tarihi:** 09.04.2012 |
|  |  | **Sayfa No:** 17/19 |
|  |  | **Onaylayan:** DB |

| | **A.1** | | **Araştırmanın açık adını belirtiniz:** Fetal distres gelişen gebelerde, anestezi tekniğinin maternal ve neonatal morbidite üzerine etkinliğinin araştırılması | | | | | | | | | | | | | | | | | --- | --- | --- | --- | --- | --- | --- | --- | --- | --- | --- | --- | --- | --- | --- | --- | --- | --- | | **B.1** | | **Araştırma protokolünün varsa kod numarasını belirtiniz:** | | | | | | | | | | | | | | | | | **B.2** | | **Araştırma protokolünün tarihini belirtiniz:** 30.05.2015 | | | | | | | | | | | | | | | | | **B.3** | | **Araştırma protokolünün versiyon numarasını belirtiniz:** | | | | | | | | | | | | | | | | | **C.1** | | | | | **Destekleyen kurum/kuruluşun adını belirtiniz:** yoktur | | | | | | | | | | | | | | **C.1.1** | | | | | **Destekleyen kurum/kuruluşun açık adresini belirtiniz:** | | | | | | | | | | | | | | **C.2** | | | | | **Varsa destekleyicinin yasal temsilcisinin adını belirtiniz:**     yoktur | | | | | | | | | | | | | | **C.2.1** | | | | | **Varsa destekleyicinin yasal temsilcisinin açık adresini belirtiniz:** | | | | | | | | | | | | | | **D.1** | | **Koordinatörün unvanı/adı/soyadını belirtiniz:** Saadet İpek Edipoğlu | | | | | | | | | | | | | | | | | **D.2** | | **Koordinatörün açık adresini belirtiniz:** Süleymaniye Kadın Doğum ve Çocuk Hastalıkları Hastanesi | | | | | | | | | | | | | | | | | **E.1** | | **Sorumlu araştırmacının unvanı/adı/soyadını belirtiniz:** Saadet İpek Edipoğlu | | | | | | | | | | | | | | | | | **E.2** | | **Sorumlu araştırmacının açık adresini belirtiniz:** Süleymaniye Kadın Doğum ve Çocuk Hastalıkları Hastanesi | | | | | | | | | | | | | | | | | **F.1** | | **Araştırmaya ülkemizde katılan merkez sayısını belirtiniz:** Tek merkez | | | | | | | | | | | | | | | | | **F.2** | | **Ülkemizdeki araştırma merkezlerinin adlarını belirtiniz:** | | | | | | | | | | | | | | | | | **G.1** | | **Araştırmaya ülkemizden alınması planlanan toplam gönüllü sayısını belirtiniz:** 100 | | | | | | | | | | | | | | | | | **H.1** | | | | **Araştırmayı tamamlayan her bir gönüllü için araştırma kurumuna ödenecek miktarı belirtiniz:**yoktur | | | | | | | | | | | | |  | | **H.1.1** | | | | **Çok merkezli araştırmalarda merkezler arasında ödeme şekli/tutarı arasında farklılık varsa merkez isimleri ile belirtiniz:** | | | | | | | | | | | | |  | | **I.1** | **Araştırmayı tamamlayan her bir gönüllü için araştırmacıya ödenecek miktarı belirtiniz:** | | | | | | | | | | | | | | | | | | **I.2** | **Çok merkezli araştırmalarda merkezler arasında farklılık varsa belirtiniz:** | | | | | | | | | | | | | | | | | | **İ.1** | | **Araştırma yerinden hizmet alımı** | | | | | | | | | **Var** | | | | | **Yok** | | | **İ.2** | | **Araştırma yeri dışından hizmet alımı** | | | | | | | | | **Var** | | | | | **Yok** | | | **J.1** | | | **Araştırma yerinden alınacak hizmetler araştırma toplam bütçesinden mi karşılanacak?** | | | | | | | | **Evet** | | | **Hayır** | | | | | **J.1.1** | | | Evet ise; alınacak hizmetleri ve miktarlarını belirtiniz: (Örneğin; 3 defa EKG, 2 defa CT gibi) | | | | | | | |  | | | | | | | | **J.2** | | | **Araştırma yeri dışından alınacak hizmetler araştırma toplam bütçesinden mi karşılanacak?** | | | | | | | | **Evet** | | | **Hayır** | | | | | **J.2.1** | | | Evet ise; alınacak hizmetleri ve miktarlarını belirtiniz: (Örneğin; 3 defa EKG, 2 defa CT gibi) | | | | | | | |  | | | | | | | | **K.1** | | | | | | | **Gönüllülere yapılacak ödemeler** | | | | | | | | | | | | **K.1.1** | | | | | | | **Gönüllülerin masraflarının geri ödenmesi şeklinde ödemeler** | | | **Var** | | | **Yok** | | | | | | **K.1.1.1** | | | | | | | Var ise; *(Ulaşım ve hafif öğle yemeği gibi)* çalışma boyunca gönüllüye ödenmesi planlanan maksimum ödeme miktarını belirtiniz: | | | | | | | | | | | | **L.1** | | | | | **Diğer ödemeler** | | | **Var** | | | | **Yok** | | | | | | | **L.1.1** | | | | | Varsa, belirtiniz: | | | | | | | | | | | | | | **M.1** | | **Araştırmanın ülkemiz için ayrılan toplam bütçesini belirtiniz:**Yoktur | | | | | | | | | | | | | | | | | **N.1** | | | | | **Gelirlerin kaynağı** | | | | | | | | | | | | | | **N.1.1** | | | | | **Destekleyici** | | | | Yoktur | | | | | | | | | | **N.1.1.1** | | | | | Belirtiniz. | | | |  | | | | | | | | | | **N.1.2** | | | | | **Araştırma fonları** | | | | Yoktur | | | | | | | | | | **N.1.2.1** | | | | | Belirtiniz. | | | |  | | | | | | | | | | **N.1.3** | | | | | **Kurum genel bütçesi** | | | | Yoktur | | | | | | | | | | **N.1.3.1** | | | | | Belirtiniz. | | | |  | | | | | | | | | | **N.1.4** | | | | | **Diğer kaynaklar** | | | | Yoktur | | | | | | | | | | **N.1.4.1** | | | | | Belirtiniz. | | | |  | | | | | | | | | | **O.1** | | | | | | **Araştırma bütçe formuna eklenen belgelerin listesi** | | | | | | | | | | | | | **O.1.1** | | | | | | **İlgili etik kurul kararı^*^**  **^*^**Etik kurul kararının aslı veya aslı gibidir onaylı örneği sunulmalıdır. | | | | | | | | |  | | | | **O.1.2** | | | | | | **Güncel imza sirküleri** | | | | | | | | |  | | | | Denenecek araştırma ürününün ücreti ve onunla ilgili olarak yapılacak test, laboratuvar vb. muayenelerin hiçbiri, varsa karşılaştırma ilacının ve/veya araştırma ürününün ücreti ile karşılaştırma için kullanılan yerleşmiş (komparatör) ilacın/araştırma ürününün kullanılışı ile ilgili test, laboratuvar vb. muayenelerinin bedelinin kamuya ait fon ve bütçelerden veya özel sağlık sigortalarından karşılanmayacağını veya kişiye ödettirilmeyeceğini, projeyi destekleyen kişi veya kuruluş tarafından ödeneceğini taahhüt ederim. | | | | | | | | | | | | | | | | | |  | Destekleyicinin veya yasal temsilcisinin el yazısıyla unvanı/adı/soyadı | Yoktur | | --- | --- | | Açık adresi |  | | Telefon numarası |  | | Faks numarası |  | | E-posta adresi |  | | Tarih (gün, ay, yıl olarak belirtiniz) |  | | İmza* |  |   *İmza sahibi imza sirkülerinde belirtilen yetkili kişi/kişiler olmalıdır.  **Ödemeler, çalışmaya alınan gönüllü sayısı, gerçekleşen vizit ve tetkik bedellerine göre yapılacaktır.  ***Bütçe, GG/AAA/YYYY tarihli koordinatör merkezin tetkik bedelleri göz önünde bulundurularak hesaplanmıştır. Destekleyici çalışma sürecince gerçekleşecek fiyat değişikliklerini ödemeyi taahhüt eder. |
| --- | --- | --- | --- | --- | --- | --- | --- | --- | --- | --- | --- | --- | --- | --- | --- | --- | --- | --- | --- | --- | --- | --- | --- | --- | --- | --- | --- | --- | --- | --- | --- | --- | --- | --- | --- | --- | --- | --- | --- | --- | --- | --- | --- | --- | --- | --- | --- | --- | --- | --- | --- | --- | --- | --- | --- | --- | --- | --- | --- | --- | --- | --- | --- | --- | --- | --- | --- | --- | --- | --- | --- | --- | --- | --- | --- | --- | --- | --- | --- | --- | --- | --- | --- | --- | --- | --- | --- | --- | --- | --- | --- | --- | --- | --- | --- | --- | --- | --- | --- | --- | --- | --- | --- | --- | --- | --- | --- | --- | --- | --- | --- | --- | --- | --- | --- | --- | --- | --- | --- | --- | --- | --- | --- | --- | --- | --- | --- | --- | --- | --- | --- | --- | --- | --- | --- | --- | --- | --- | --- | --- | --- | --- | --- | --- | --- | --- | --- | --- | --- | --- | --- | --- | --- | --- | --- | --- | --- | --- | --- | --- | --- | --- | --- | --- | --- | --- | --- | --- | --- | --- | --- | --- | --- | --- | --- | --- | --- | --- | --- | --- | --- | --- | --- | --- | --- | --- | --- | --- | --- | --- | --- | --- | --- | --- | --- | --- | --- | --- | --- | --- | --- | --- | --- | --- | --- | --- | --- | --- | --- | --- | --- | --- | --- | --- | --- | --- | --- | --- | --- | --- | --- | --- | --- | --- | --- | --- | --- | --- | --- | --- | --- | --- | --- | --- | --- | --- | --- | --- | --- | --- | --- | --- | --- | --- | --- | --- | --- | --- | --- | --- | --- | --- | --- | --- | --- | --- | --- | --- | --- | --- | --- | --- | --- | --- | --- | --- | --- | --- | --- | --- | --- | --- | --- | --- | --- | --- | --- | --- | --- | --- | --- | --- | --- | --- | --- | --- | --- | --- | --- | --- | --- | --- | --- | --- | --- | --- | --- | --- | --- | --- | --- | --- | --- | --- | --- | --- | --- | --- | --- | --- | --- | --- | --- | --- | --- | --- | --- | --- | --- | --- | --- | --- | --- | --- | --- | --- | --- | --- | --- | --- | --- | --- | --- | --- | --- | --- | --- | --- | --- | --- | --- | --- | --- | --- | --- | --- | --- | --- | --- | --- | --- | --- | --- | --- | --- | --- | --- | --- | --- | --- | --- | --- | --- | --- | --- | --- | --- | --- | --- | --- | --- | --- | --- | --- | --- | --- | --- | --- | --- | --- | --- | --- | --- | --- | --- | --- | --- | --- | --- | --- | --- | --- | --- | --- | --- | --- | --- | --- | --- | --- | --- | --- | --- | --- | --- | --- | --- | --- | --- | --- | --- | --- | --- | --- | --- | --- | --- | --- | --- | --- | --- | --- | --- | --- | --- | --- | --- | --- | --- | --- | --- | --- | --- | --- | --- | --- | --- | --- | --- | --- | --- | --- | --- | --- | --- | --- | --- | --- | --- | --- | --- | --- | --- | --- | --- | --- | --- | --- | --- | --- | --- | --- | --- | --- | --- | --- | --- | --- | --- | --- | --- | --- | --- | --- | --- | --- | --- | --- | --- | --- | --- | --- | --- | --- | --- | --- | --- | --- | --- | --- | --- | --- | --- | --- | --- | --- | --- | --- | --- | --- | --- | --- | --- | --- | --- | --- | --- | --- | --- | --- | --- | --- | --- | --- | --- | --- | --- | --- | --- | --- | --- | --- | --- | --- | --- | --- | --- | --- | --- | --- | --- | --- | --- | --- | --- | --- | --- | --- | --- | --- | --- | --- | --- | --- | --- | --- | --- | --- | --- | --- | --- | --- | --- | --- | --- | --- | --- | --- | --- | --- | --- | --- | --- | --- | --- | --- | --- | --- | --- | --- | --- | --- | --- | --- | --- | --- | --- | --- | --- | --- | --- | --- | --- | --- | --- | --- | --- | --- | --- | --- | --- | --- | --- | --- | --- | --- | --- | --- | --- | --- | --- | --- | --- | --- | --- | --- | --- | --- | --- | --- | --- | --- | --- | --- | --- | --- | --- | --- | --- | --- | --- | --- | --- | --- | --- | --- | --- | --- | --- | --- | --- | --- | --- | --- | --- | --- | --- | --- | --- | --- | --- | --- | --- | --- | --- | --- | --- | --- | --- | --- | --- | --- | --- | --- | --- | --- | --- | --- | --- | --- | --- | --- | --- | --- | --- | --- | --- | --- | --- | --- | --- | --- | --- | --- | --- | --- | --- | --- | --- | --- | --- | --- | --- | --- | --- | --- | --- | --- | --- | --- | --- | --- | --- | --- | --- | --- | --- | --- | --- | --- | --- | --- | --- | --- | --- | --- | --- | --- | --- | --- | --- | --- | --- | --- | --- | --- | --- | --- | --- | --- | --- | --- | --- | --- | --- | --- | --- | --- | --- | --- | --- | --- | --- | --- | --- | --- | --- | --- | --- | --- | --- | --- | --- | --- | --- | --- | --- | --- | --- | --- | --- | --- | --- | --- | --- | --- | --- | --- | --- | --- | --- | --- | --- | --- | --- | --- | --- | --- | --- | --- | --- | --- | --- | --- | --- | --- | --- | --- | --- | --- | --- | --- | --- | --- | --- | --- | --- | --- | --- | --- | --- | --- | --- | --- | --- | --- | --- | --- | --- | --- | --- | --- | --- | --- | --- | --- |

**ASGARİ BİLGİLENDİRİLMİŞ GÖNÜLLÜ OLUR FORMU**

**Çalışmamızın yöntemi retrospektif olduğu ve sadece dosya taraması yapılacağı için bilgilendirilmiş gönüllü onam formu doldurmaya gerek yoktur.**

1. Araştırmaya ilk gönüllünün katılımından son gönüllüye yapılan son ziyarete kadar. [↑](#footnote-ref-2)
2. Bu bölümde ürün adı veya ürün kodu veya ürünün ticari ismi yazılmalıdır.

   ^3^ Bu bölümde ürünün ruhsat durumu hakkında bilgiler yer almaktadır.

   [↑](#footnote-ref-3)
