# Supplementary material for: Effect of anaesthetic technique on neonatal morbidity in emergency caesarean section for foetal distress
Source: PLoS One. 2018 Nov 16;13(11):e0207388. doi: 10.1371/journal.pone.0207388 (PMC6239306; doi:10.1371/journal.pone.0207388)
Supplement: S3 File — (PDF) [file pone.0207388.s004.pdf]

BMC
Part of Springer Nature

ISRCTN registry

Search
Advanced Search

View all studies | Why register? | Register your study
ipek edipoglu | My Account | Logout

ISRCTN15181117 https://doi.org/10.1186/ISRCTN15181117

The effect of anesthetic technique on neonatal morbidity in emergent cesarean section for fetal distress

Condition category
Pregnancy and Childbirth

Date applied
12/12/2015

Date assigned
15/02/2016

Last edited
15/02/2016

Prospective/Retrospective
Retrospectively registered

Overall trial status
Completed

Recruitment status
No longer recruiting

Plain English Summary

Background and study aims
An emergent (or emergency) caesarean section is one of the biggest challenges faced by an obstetric anaesthetist. As the outcome for both mother and unborn baby depends on the coordination and vigilance of the obstetrician and anaesthetist, the decision on what anesthetic technique to use can be of paramount importance. Current guidelines suggest regional (local) anaesthesia should be given in preference to general anaesthesia for elective (pre-planned) caesarean sections. However, for an emergency caesarean section, there may not be enough time to give a regional anaesthetic. Evidence does suggest that regional anaesthesia should be chosen in preference to general anaesthesia if there is time. However, there are few published studies looking at the neonatal morbidity (disease causing) for emergency caesareans where the baby has been identified as being in distress (foetal distress). In this study, neonatal morbidity will be compared for emergency caesarean sections carried out using regional anaesthesia compared with general anaesthesia.

Who can participate?
Women aged 18-45 in labour where their unborn baby is showing signs of distress.

What does the study involve?
For each patient in the study, the senior anaesthetist decides the type of anaesthesia according to both national guidelines and patients' approval. The patients are divided into two groups, general anaesthesia group (group g) or regional anaesthesia group (group r). Heart rate, arterial tension pressure and demographic data are all recorded for each patient. Once the baby is born, APGAR scores (a way of summarising the health of a newborn baby) are recorded one minute after birth and then again at 3 minutes and 5 minutes after birth. If the babies condition is critical and there are umbilical blood gases (suggesting foetal distress) this data is also recorded. After the operation , the neonates are followed up until their discharge from hospital. Any morbid (disease-causing) conditions are also recorded.

What are the possible benefits and risks of participating?
There is no risk for the participants because it is an observational study.

Where is the study run from?
Suleymaniye Birth and Women's Health Education and Research Hospital, Istanbul (Turkey)

When is the study starting and how long is it expected to run for?
July 2015 to December 2015

Who is funding the study?
Suleymaniye Birth and Women's Health Education and Research Hospital, Istanbul (Turkey)

Who is the main contact?
Dr Ipek Saadet Edipoglu

Trial website

Contact information

Type
Scientific

Primary contact
Dr Ipek Saadet Edipoglu

ORCID ID

Contact details
Suleymaniye Birth And Women's Health Education And Research Hospital
Telsiz Mah. Balıklı Kazlıcesme Yolu No:1 Zeytinburnu
Istanbul
34116
Turkey

Additional identifiers

EudraCT number

ClinicalTrials.gov number

Protocol/serial number
N/A

Study information

Scientific title
The effect of anaesthetic technique on neonatal morbidity in emergent caesarean section for fetal distress: a prospective observational study

Acronym

Study hypothesis
To investigate the effect of anesthetic technique on neonatal morbidity in emergent cesareans by observing if administering regional anesthesia or general anesthesia influences the morbidity of the neonate.

Ethics approval
Research Hospital Clinical Studies Ethical Committee, 29/06/2015, ref: 2015/127

Study design
Single centered prospective observational study.

Primary study design
Observational

Secondary study design
Cohort study

Trial setting
Hospitals

Trial type
Treatment

Patient information sheet
Not available in web format, please use contact details to request a participant information sheet

Condition
Fetal distress and anesthetic technique

Intervention
We are going to observe emergent cesarean sections which are diagnosed as fetal distress. We are not going to intervene regarding the patients anesthetic choice. Regional anesthesia or general anesthesia will be applied according to the patients indication. The consultant anesthesiologist makes the decision about the type of anesthesia for emergent cesarean sections according to our national guidelines in our hospital. We are just going to observe and divide them into two groups: general anesthesia group and regional anesthesia group. The following will be recorded in all cases:
1. All patients peri-operative heart rate and mean arterial pressures
2. All neonates APGAR scores at 1, 3 and 5 minutes
3. The need for mechanical ventilation will also be recorded.
4. All morbid conditions

Intervention type
Procedure/Surgery

Phase

Drug names

Primary outcome measure
Neonatal morbidity, measured with APGAR scores in 1st, 3rd, 5th minutes, and umbilical blood gas (if indicated). After the operation we will follow neonates until discharge and record any morbid conditions. We define morbidity as 5-minute Apgar score (APGAR5) <7, any need for mechanical ventilation, any neonatal intensive care unit entrance and any respiratory insufficiency symptoms.

Secondary outcome measures

Morbidity of the mother if administering regional anesthesia compared to general anesthesia

Overall trial start date

01/07/2015

Overall trial end date

06/12/2015

Reason abandoned (if study stopped)

Eligibility

Participant inclusion criteria

1. Female
2. Age between 18-45
3. BMI<40
4. Patients diagnosed as being in fetal distress.
5. Patients without neurological diseases
6. Patients consenting to join the study

Participant type

Patient

Age group

Adult

Gender

Female

Target number of participants

60 patients will be included.

Participant exclusion criteria

1. Age less than 18 and older than 45
2. BMI>40
3. Patients that refuse to join study

Recruitment start date

01/07/2015

Recruitment end date

06/12/2015

Locations

Countries of recruitment

Turkey

Trial participating centre

Suleymaniye Birth And Women's Health Education And Research Hospital
Telsiz Mah. Balıklı Kazlıcesme Yolu No:1 Zeytinburnu
Istanbul
34116
Turkey

Sponsor information

Organisation

Suleymaniye Birth And Women's Health Education And Research Hospital

Sponsor details

Telsiz Mah. Balıklı Kazlıcesme Yolu No:1 Zeytinburnu
Istanbul
34116
Turkey

Sponsor type

Government

Website

Funders

Funder type

Hospital/treatment centre

Funder name

Suleymaniye Birth And Women's Health Education And Research Hospital (Turkey)

Alternative name(s)

Funding Body Type

Funding Body Subtype

Location

Results and Publications

Publication and dissemination plan

Intention to publish date

01/02/2016

Participant level data

To be made available at a later date

Basic results (scientific)

Publication list

Publication citations

Additional files

Editorial Notes

About
Governance
Why register?
Search tips
Resources
FAQs
Follow us on Twitter
Publish with BioMed Central
Contact us

© 2018 BioMed Central Ltd unless otherwise stated. Part of Springer Nature.

Terms & Conditions and Privacy Policy

SPRINGER NATURE
